# Changes in Efficacy Indicators for Adalimumab Biosimilar Candidate (HS016) for the Treatment of Active Ankylosing Spondylitis at Various Time Points

**DOI:** 10.3389/fphar.2020.606497

**Published:** 2020-12-07

**Authors:** Jinmei Su, Mengtao Li, Lan He, Dongbao Zhao, Weiguo Wan, Yi Liu, Jianhua Xu, Jian Xu, Huaxiang Liu, Lindi Jiang, Huaxiang Wu, Xiaoxia Zuo, Cibo Huang, Xiumei Liu, Fen Li, Zhiyi Zhang, Xiangyuan Liu, Lingli Dong, Tianwang Li, Haiying Chen, Jingyang Li, Dongyi He, Xin Lu, Anbin Huang, Yi Tao, Yanyan Wang, Zhuoli Zhang, Wei Wei, Xiaofeng Li, Xiaofeng Zeng

**Affiliations:** ^1^ Department of Rheumatology, Peking Union Medical College Hospital, Peking Union Medical College and Chinese Academy of Medical Sciences, National Clinical Research Center for Immunologic Diseases, Ministry of Science and Technology, Key Laboratory of Rheumatology and Clinical Immunology, Ministry of Education, Beijing, China; ^2^ Department of Rheumatology and Immunology, The First Affiliated Hospital of Xi’an Jiaotong University, Xi’an, China; ^3^ Department of Rheumatology, Changhai Hospital, Shanghai, China; ^4^ Department of Rheumatology, Huashan Hospital, Fudan University, Shanghai, China; ^5^ Department of Rheumatology, West China Hospital, Sichuan University, Chengdu, China; ^6^ Department of Rheumatology, The First Affiliated Hospital of Anhui Medical University, Hefei, China; ^7^ Department of Rheumatology, The First Affiliated Hospital of Kunming Medical University, Kunming, China; ^8^ Department of Rheumatology, Qilu Hospital of Shandong University, Jinan, China; ^9^ Department of Rheumatology, Zhongshan Hospital, Fudan University, Shanghai, China; ^10^ Department of Rheumatology, The Second Affiliated Hospital of Zhejiang University School of Medicine, Hangzhou, China; ^11^ Department of Rheumatology, Xiangya Hospital, Central South University, Changsha, China; ^12^ Department of Rheumatology, Beijing Hospital, Beijing, China; ^13^ Department of Rheumatology, The First Affiliated Hospital of Shanxi Medical University, Taiyuan, China; ^14^ Department of Rheumatology, The Second Xiangya Hospital of Central South University, Changsha, China; ^15^ Department of Rheumatology, The First Affiliated Hospital of Harbin Medical University, Harbin, China; ^16^ Department of Rheumatology, Peking University Third Hospital, Beijing, China; ^17^ Department of Rheumatology, Tongji Hospital, Tongji Medical College of Huazhong University of Science and Technology, Wuhan, China; ^18^ Department of Rheumatology, Guangdong Second Provincial General Hospital, Guangzhou, China; ^19^ Department of Rheumatology, The Third Hospital of Hebei Medical University, Shijiazhuang, China; ^20^ Department of Rheumatology, Zhuzhou Central Hospital, Zhuzhou, China; ^21^ Department of Rheumatology, Shanghai Guanghua Hospital of Integrated Traditional Chinese and Western Medicine, Shanghai, China; ^22^ Department of Rheumatology, China-Japan Friendship Hospital, Beijing, China; ^23^ Department of Rheumatology, Union Hospital, Tongji Medical College of Huazhong University of Science and Technology, Wuhan, China; ^24^ Department of Rheumatology, The Second Affiliated Hospital of Guangzhou Medical University, Guangzhou, China; ^25^ Department of Rheumatology, Jiangsu Province Hospital, Nanjing, China; ^26^ Department of Rheumatology, Peking University First Hospital, Beijing, China; ^27^ Department of Rheumatology, Tianjin Medical University General Hospital, Tianjin, China; ^28^ Department of Rheumatology, The Second Affiliated Hospital of Shanxi Medical University, Taiyuan, China

**Keywords:** ankylosing spondylitis, adalimumab, HS016, subanalysis, phase III clinical trial

## Abstract

**Objectives:** A phase III, 24-weeks Chinese clinical trial demonstrated that efficacy and safety outcomes of treatments with 40 mg/0.8 ml HS016 (*n* = 416) or adalimumab (*n* = 232) for active ankylosing spondylitis (AS) patients was comparable. In the present study, a subanalysis of the clinical trial was conducted to determine whether also individual efficacy indicators were comparable between HS016 and adalimumab.

**Methods:** The individual efficacy indicators total and nocturnal back pain, global assessment of disease activity, swollen joint count, Maastricht AS Enthesitis Score, Bath AS Disease Activity Index, Bath AS Functional Index, Bath AS Metrology Index and chest expansion, were assessed at baseline and every 2 weeks during the treatment period.

**Results:** This subanalysis revealed no significant difference between the patient groups treated with HS016 or adalimumab for any individual efficacy indicator investigated at any time point (all *p* > 0.05) beside faster total back pain score improvements in the adalimumab group on week 10, 12 and 22, which became equal at week 24. Among these indicators, chest expansion showed a significant increase at each time point compared with baseline, whereas all other efficacy indicators showed significant decreases compared with baseline at each time point (all *p* < 0.05). All efficacy indicators had increased or decreased rapidly by week 2, and the values continued to increase/decrease up to week 12, with subsequent smaller changes thereafter up to week 24 of treatment.

**Conclusion:** The response trajectory of most individual efficacy indicators was comparable between HS016 and adalimumab at each time point during the 24 weeks of the trial.

**Clinical Trial Registration:** http://www.chictr.org.cn/showproj.aspx?proj=37910, identifier [ChiCTR1900022520]

## Introduction

Ankylosing spondylitis (AS), a chronic inflammatory disease affecting the skeleton, causes inflammatory pain in the back and structural and functional impairments, that mainly affects males (Braun and Sieper, 2007; de Winter et al., 2016).

Nonsteroidal anti-inflammatory drugs (NSAIDs) and tumor necrosis factor (TNF-α) blockers are commonly used treatments for AS; if the lesions affect the hip joint or there is a spinal deformity, surgery may also be performed (Ward et al., 2016). Although NSAIDs are the recommended first-line pharmacological treatment for AS, approximately 50% of people with this disease have reported that NSAIDs alone do not adequately alleviate their symptoms. Furthermore, NSAIDs produce significant gastrointestinal toxicity and potential adverse cardiovascular affects (Ward and Kuzis, 2002). TNF-α inhibitors are effective when used to treat rheumatoid arthritis and various other autoimmune inflammatory disease states where TNF-α is involved in the pathogenesis (Lim et al., 2018). This class of drug is currently used to treat AS patients who exhibit extra-articular symptoms and fail to response to NSAIDs (Ward et al., 2016). Unfortunately, TNF-α blockers are expensive and this has limited their use in patients with AS on modest salaries and who cannot afford healthcare insurance (Pelechas et al., 2018). HS016, a biosimilar candidate of adalimumab (150 kD) (Mounach and El Maghraoui, 2014), which is a recombinant human monoclonal antibody (approximately 148 kD) that interacts with TNF-α, preventing it from exerting its inflammatory actions in AS patients (van der Heijde et al., 2006). After a successful phase I evaluation (Cao et al., 2020), the results from a multicenter, phase III clinical trial demonstrated that HS016 and adalimumab had efficacy and safety profiles when administered for a treatment period of 24 weeks (Su et al., 2020).

An increase in the number of patients that exhibited about a 20% improvement in symptoms from baseline, according to Assessment of Spondyloarthritis International Society criteria (ASAS20), was found during the second week of therapy for the HS016 (46.4%) and adalimumab (47.4%) groups, which continued up to week 24. No differences were found in the ASAS20 response rates in either treatment group at week 12 (79.6%, 331/416 vs. 81.0%, 188/232) or week 24 (87.5%, 364/416 vs. 90.1%, 209/232) (Su et al., 2020). However, even though the efficacy of HS016 and adalimumab was equivalent at week 24 for the treatment of active AS, the efficacy was determined using composite endpoints, which included several individual indicators and the changes in the response over time for individual efficacy indicators. Whether the responses were comparable between adalimumab and HS016 at all time points remains unknown.

Therefore, the aim of the present research was to establish the direction and timing of individual efficacy indicator responses and to determine whether the response to HS016 and adalimumab was similar during 24 weeks of treatment.

## Methods

### Study Design and Patients

The design of the clinical trial has been previously described (Su et al., 2020). Briefly, patients were enrolled into an HS016 group (416 patients) or an adalimumab group (232 patients) in 28 centers across China (Supplementary Figure S1). The enrolled patients (age range: 18–65 years) had active AS that fulfilled the 1984 modified New York classification criteria (van der Linden et al., 1984) and had not received other TNF-α inhibitors within 12 weeks before randomization. Additional information on the inclusion and exclusion criteria has been reported elsewhere (Su et al., 2020). Eligible patients were randomized using a double-blind protocol and received a subcutaneous injection of HS016 or adalimumab at a dose of 40 mg/0.8 ml once on alternate 2 weeks for a 24-week treatment period. The random and drug numbers for patients were generated by the Central Random System (IWRS), but did not provide treatment information. The evaluation for individual efficacy indicators was carried out by the investigators, but due to the double-blind design, the investigators and patients were blinded to the trial grouping.

The study was registered with the Chinese Clinical Trial Registry (No. ChiCTR1900022520) and carried out by strictly following the guidelines of the Good Clinical Practice and Provisions for Drug Registration of the National Medical Products Administration (NMPA). An ethics committee at every participating center granted approval of the study protocols and reviewed all amendments. All patients provided signed informed consent before they were enrolled in the trial.

### Efficacy Indicator Assessments

According to the outcome measures for disease-controlling antirheumatic treatments (DC-ART) (van der Heijde et al., 1999), the individual indicators relevant for the determination of efficacy were nocturnal back pain and total back pain, the swollen joints count (SJC), Maastricht AS Enthesitis Score (MASES), patient global assessment (PaGA) and physician global assessment (PhGA) of the activity of AS, Bath AS Disease Activity Index (BASDAI), Bath AS Functional Index (BASFI), linear Bath AS Metrology Index (BASMI_lin_) and chest expansion at each time point during the 24 weeks of treatment. We did not include the ASDAS or severity of morning stiffness, as these data were already reported in a previous study (Su et al., 2020). These indicators were evaluated at baseline and at 2-week intervals throughout the entire treatment period in the trial.

A 0–10 cm numerical rating scale (NRS) was used to rate the degree of pain (0 denoted as no significant pain and a score of 10 the worst pain). The factors assessed were nocturnal back pain and total back pain, the PaGA and PhGA of activity of AS, BASDAI and BASFI. The evaluation of swelling in 46 joints (each joint scoring 0, 1, 2 or 3) was performed by a clinician. The MASES (range 0–13) was evaluated as the sum of 13 entheseal sites scored dichotomously as 0 (enthesis absent) or 1 (enthesis present). The BASDAI was comprised of six questions on joint pain/swelling, fatigue, spinal pain, localized areas of tenderness, the severity of stiffness in the morning and the duration of morning stiffness. These questions are relevant to the five major clinical features seen in active AS patients (Garrett et al., 1994). The BASMI_lin_ score was determined as the mean score for the following items: lateral spinal flexion (both the left and right sides); tragus to the wall distances (both sides); lumbar flexion; the maximal intermalleolar distance; and the degree of cervical rotation (measured with a goniometer). Chest expansion (cm) was measured as the maximum difference in the thoracic circumference between one deep inspiration and exhalation.

### Statistical Analysis

SAS (version 9.2) was employed for statistical analysis. The population used for analysis of the individual efficacy indicators in the two groups was the full analysis set (FAS) i.e., involving all patients who were given one or more doses of the trial drugs.

The last observation carried forward method was employed to input any missing data in a given sample for use in the covariance model and analysis of changes after treatment. Two-way Analysis of Variance was employed to compare the individual efficacy indicators at each 2-week time points between HS016 and adalimumab groups. In addition, Greenhouse-Geisser correction is used in the test of hypotheses for within subject effects. An independent group *t*-test was employed to compare the changes of individual efficacy indicators from baseline at 2, 4, 6, 8, 10, 12, 14, 16, 18, 20, 22, and 24 weeks after treatment initiation between the adalimumab and HS016 groups. The significance of changes in the efficacy indicator values at each treatment time point compared with the baseline was evaluated with a paired *t*-test in each adalimumab or HS016 group. A *p-*value (two-sided) <0.05 was deemed to be a significant result.

## Results

### Individual Efficacy Indicators at Baseline

The baseline individual efficacy indicators are shown in Table 1 for the two groups. The data clearly shows that there were no significant differences between any of the efficacy indicators investigated at baseline (all *p* > 0.05).

**TABLE 1:** Individual efficacy indicators at baseline in the HS016 and adalimumab groups.

Indicator^a^	HS016 (*n* = 416)	Adalimumab (*n* = 232)	*p*-value
Total back pain score (0–10 cm NRS)	6.78 ± 1.61	7.00 ± 1.60	0.092
Nocturnal back pain score (0–10 cm NRS)	6.66 ± 1.80	6.90 ± 1.94	0.114
SJC (46 joints)	0.24 ± 1.00	0.37 ± 2.30	0.312
MASES (range 0–13)	1.58 ± 2.26	1.76 ± 2.41	0.355
PaGA of disease activity (0–10 cm NRS)	6.80 ± 1.58	6.96 ±1.64	0.212
PhGA of disease activity (0–10 cm NRS)	6.28 ± 1.46	6.37 ± 1.47	0.452
BASDAI	6.24 ± 1.30	6.33 ± 1.38	0.401
BASFI	4.57 ± 2.30	4.71 ± 2.37	0.467
BASMI_lin_	1.26 ± 1.66	1.13 ± 1.62	0.311
BASMI_lin__lateral spinal flexion	5.40 ± 2.67	5.28 ± 2.75	0.568
BASMI_lin__tragus to wall distance	1.90 ± 1.70	1.70 ± 1.47	0.128
BASMI_lin__lumbar flexion	−8.04 ± 2.82	−8.37 ± 2.68	0.148
BASMI_lin__maximal intermalleolar distance	2.55 ± 2.27	2.65 ± 2.32	0.576
BASMI_lin__cervical rotation	4.49 ± 2.13	4.37 ± 1.95	0.485
Chest expansion (cm)	3.60 ± 1.99	3.59 ± 1.84	0.940

BASDAI, Bath ankylosing spondylitis disease activity index; BASFI, Bath ankylosing spondylitis functional index; BASMI_lin_, linear Bath ankylosing spondylitis metrology index; MASES, Maastricht ankylosing spondylitis enthesitis score; NRS, numerical rating scale; PaGA, Patient global assessment; PhGA, Physician global assessment; SJC, swollen joint count.

a All data are presented as the mean ± SD.

### Pain Score Trends

**FIGURE 1:**
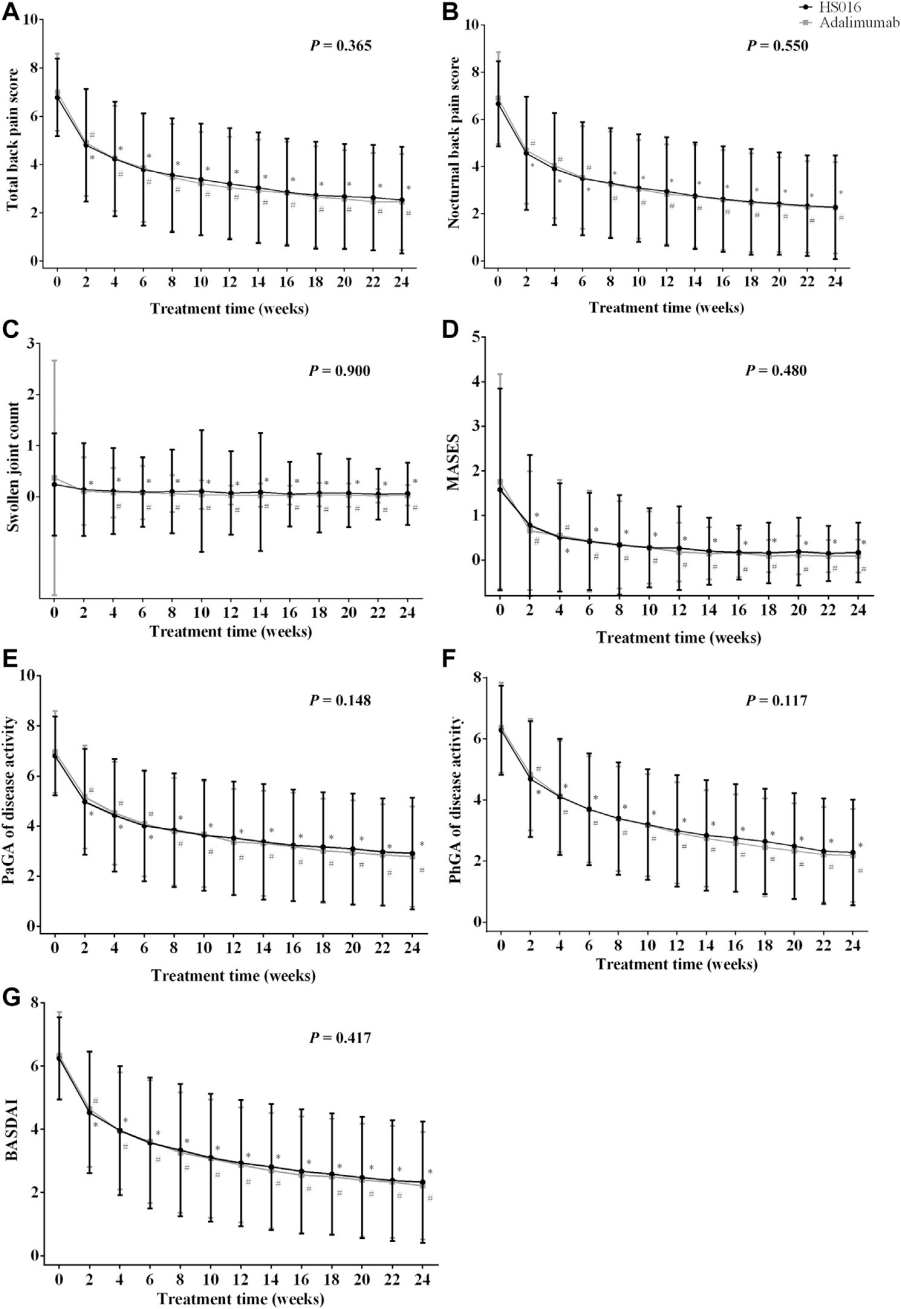
**(A)** Total back pain, **(B)** nocturnal back pain, **(C)** swollen joint count, **(D)** MASES, **(E)** PaGA, **(F)** PhGA, and **(G)** BASDAI values at each time point during the 24-week treatment period in patients treated with HS016 or adalimumab. *p < 0.05, compared with baseline in the HS016 group; ^#^ *p* < 0.05, compared with baseline in the adalimumab group. BASDAI, Bath ankylosing spondylitis disease activity index; MASES, Maastricht ankylosing spondylitis enthesitis score; PaGA, Patient global assessment; PhGA, Physician global assessment; SJC, swollen joint count.

In Table 2, only efficacy indicator changes at weeks 2, 12 and, 24 are listed, but measurements were made every 2 weeks until 24 weeks after the commencement of treatment. Rapid decreases from baseline in the total back pain score (−1.98 ± 2.12, 29.20% and −2.10 ± 2.03, 30.00%) and nocturnal back pain score (−2.10 ± 2.27, 31.53% and −2.21 ± 2.07, 32.03%) occurred in the HS016 and adalimumab groups at week 2 (all *p* < 0.05), and these changes were broadly similar between the two groups (all *p* > 0.05) (Table 2). The decreasing trends for total and nocturnal back pain scores continued up to week 12 (HS016 group: −52.80 and −55.56%; adalimumab group: −56.57; and −58.99%, respectively), at which point they changed to slightly decreasing trends that continued throughout the 24 weeks of treatment (HS016 group: −62.68 and −65.77; adalimumab group: −65.14 and −67.10%) with a significant difference in each time point from baseline for each group (all *p* < 0.05). These two pain assessment scores were comparable in the two groups at each time point (*p* = 0.365 and *p* = 0.550) (Figures 1A,B). However, with regard to the total back pain score, we found that decreases from baseline to week 10 (−3.40 ± 2.30 vs. −3.80 ± 2.32, *p* = 0.038), week 12 (−3.58 ± 2.28 vs. −3.96 ± 2.27, *p* = 0.040) and week 22 (−4.15 ± 2.34 vs. −4.54 ± 2.25, *p* = 0.038) were significantly smaller in the HS016 group compared to the adalimumab group, and the data indicated that reductions in total back pain scores at weeks 10, 12, and 22 were greater in the adalimumab treated group of patients.

**TABLE 2:** Mean changes from baseline in individual efficacy indicators.

Indicators^a^	HS016 (*n* = 416)	Adalimumab (*n* = 232)	*p*-value
∆Total back pain score (0–10 cm NRS)			
Week 2	–1.98 ± 2.12	–2.10 ± 2.03	0.494
Week 10^b^	–3.40 ± 2.30	–3.80 ± 2.32	0.038
Week 12	–3.58 ± 2.28	–3.96 ± 2.27	0.040
Week 22^b^	–4.15 ± 2.34	–4.54 ± 2.25	0.038
Week 24	–4.25 ± 2.32	–4.56 ± 2.26	0.111
∆Nocturnal back pain score (0–10 cm NRS)			
Week 2	–2.10 ± 2.27	–2.21 ± 2.07	0.548
Week 12	–3.70 ± 2.43	–4.07 ± 2.41	0.065
Week 24	–4.38 ± 2.41	–4.63 ± 2.38	0.193
∆SJC (46 joints)			
Week 2	–0.10 ± 0.87	–0.26 ± 2.04	0.159
Week 12	–0.16 ± 1.01	–0.34 ± 2.26	0.184
Week 24	–0.18 ± 0.94	–0.34 ± 2.30	0.200
∆MASES (range 0–13)			
Week 2	–0.80 ± 1.86	–1.10 ± 1.94	0.054
Week 12	–1.32 ± 2.08	–1.58 ± 2.29	0.142
Week 24	–1.41 ± 2.19	–1.67 ± 2.40	0.162
∆PaGA of disease activity (0–10 cm NRS)			
Week 2	–1.83 ± 1.98	–1.80 ± 2.03	0.865
Week 12	–3.28 ± 2.24	–3.60 ± 2.42	0.088
Week 24	–3.89 ± 2.37	–4.18 ± 2.38	0.137
∆PhGA of disease activity (0–10 cm NRS)			
Week 2	–1.59 ± 1.49	–1.53 ± 1.49	0.628
Week 12	–3.28 ± 1.74	–3.45 ± 1.74	0.243
Week 24	–3.99 ± 1.75	–4.18 ± 1.84	0.190
∆BASDAI (0–10 cm NRS)			
Week 2	–1.71 ± 1.64	–1.68 ± 1.54	0.843
Week 12	–3.31 ± 1.94	–3.46 ± 1.97	0.353
Week 24	–3.91 ± 1.98	–4.12 ± 1.94	0.202
∆BASFI (0–10 cm NRS)			
Week 2	–1.05 ± 1.73	–1.05 ± 1.56	0.974
Week 12	–2.20 ± 2.08	–2.27 ± 2.00	0.706
Week 24	–2.59 ± 2.23	–2.66 ± 2.11	0.688
∆BASMI_lin_			
Week 2	–0.37 ± 0.55	–0.33 ± 0.67	0.455
Week 12	–0.75 ± 0.79	–0.69 ± 0.82	0.315
Week 24	–0.86 ± 0.91	–0.85 ± 0.91	0.948
∆Chest expansion (cm)			
Week 2	0.39 ± 1.64	0.37 ± 1.27	0.922
Week 12	0.62 ± 1.88	0.59 ± 1.82	0.839
Week 24	0.88 ± 2.10	0.78 ± 1.88	0.566

BASDAI, Bath ankylosing spondylitis disease activity index; BASFI, Bath ankylosing spondylitis functional index; BASMI_lin_, linear Bath ankylosing spondylitis metrology index; MASES, Maastricht ankylosing spondylitis enthesitis score; NRS, numerical rating scale; PaGA, Patient global assessment; PhGA, Physician global assessment; SJC, swollen joint count.

a All data are presented as the means ± SDs, ∆ = values at each point-baseline.

b This data have been added since they comprise the only significant differences between the groups within other time points than week 2, 12, and 24. An independent group *t*-test was utilized to identify any changes from baseline to week 2, week 12, and week 24 between the two groups. A two-sided *p*-value <0.05 was deemed significant.

### Swollen Joints Count and Maastricht Ankylosing Spondylitis Enthesitis Score Trends

After 2 weeks of treatment, even though there were declining trends in SJC in both the HS016 (−0.10 ± 0.87; −41.67%) and adalimumab (−0.26 ± 2.04; −70.27%) groups compared to baseline, only the difference in the HS016 group was significant (*p* = 0.024); however, the SJC changes from baseline in the two groups at 2-week were not statistically significantly (*p* = 0.159) (Table 2). But a significant decrease in SJC compared with baseline was found in both groups from weeks 4 to 24 (all *p* < 0.05) (Figure 1C). The declining trend in SJC continued until the 12th week of treatment (decreased rate from baseline: −66.67 and −91.89%), and then SJC had a lesser decreasing trend that lasted until the end of the treatment period in both groups (decreased rate from baseline: −75.00 and −91.89%).

Regarding the MASES, after 2 weeks of treatment, a significant decrease was observed from 1.58 ± 2.26 to 0.78 ± 1.58 (−50.63%) in the HS016 group and from 1.76 ± 2.41 to 0.66 ± 1.33 (−62.60%) in the adalimumab group (Table 2). The declining trends continued up to week 12 of treatment, with differences of −1.32 ± 2.08 (−83.54%) and −1.58 ± 2.29 (−89.77%) from baseline in the two groups, respectively. The rate of decline decreased from week 12 to week 24 compared to that from baseline to week 12 (Figure 1D); the differences from baseline at week 24 were −1.41 ± 2.19 (−89.24%) and −1.67 ± 2.40 (−94.89%) for the HS016 group and the adalimumab group, respectively (Table 2).

No differences in SJC or MASES values were found for the HS016 and adalimumab groups at all time points investigated (*p* = 0.900 and *p* = 0.480, Figures 1C,D). Similarly, no significant difference was found for changes in SJC or MASES from baseline in the two groups (all *p* > 0.05).

### Patient Global Assessment and Physician Global Assessment of Disease Activity and Bath Ankylosing Spondylitis Disease Activity Index Score Trends

The PaGA, PhGA and BASDAI were measured using a 0–10 cm NRS to evaluate disease activity. The PaGA (4.97 ± 2.12; 5.16 ± 2.05) and PhGA (4.68 ± 1.90; 4.83 ± 1.82) at week two in both groups were substantially decreased compared with baseline (HS016 and adalimumab: −26.91 and −25.86%; −25.32 and −24.02%, respectively) (all *p* < 0.05). Following week two of treatment, these assessment scores decreased at all subsequent time points, with the decline slowing from week 12 (HS016 and adalimumab: −48.24 and −51.72% PaGA; −52.23 and −54.16% PhGA, respectively) to week 24 (HS016 and adalimumab: −57.21 and −60.06%; −63.54 and −65.62%) (Figures 1E,F). The changes in the PaGA and PhGA scores from baseline to week 12 and week 24 also showed slowly declining trends in both groups (Table 2).

The BASDAI score decreased from 6.24 ± 1.30 to 4.53 ± 1.92 (−27.4%) in the HS016 group and from 6.33 ± 1.38 to 4.65 ± 1.83 (−26.54%) in the adalimumab group at week 2 and continued to decrease up to end of the treatment period, although the decline was more pronounced up to week 12 (2.93 ± 2.00 and 2.87 ± 1.82 BASDAI score; −53.04 and −54.66% difference, in the HS016 and adalimumab groups, respectively), than from week 12 to week 24 (2.33 ± 1.92 and 2.21 ± 1.70 BASDAI score, −62.66% and −65.09% difference from baseline, in the HS016 and adalimumab groups, respectively) (Figure 1G).

In summary, no differences in the PaGA and PhGA of disease activity or BASDAI scores were found between the HS016 and adalimumab groups at any time point (*p* = 0.148, *p* = 0.117 and *p* = 0.417), but decreases from baseline in these three indicators were observed in both groups (all *p* < 0.05) (Figures 1E–G).

### Bath Ankylosing Spondylitis Functional Index Score Trends

**FIGURE 2:**
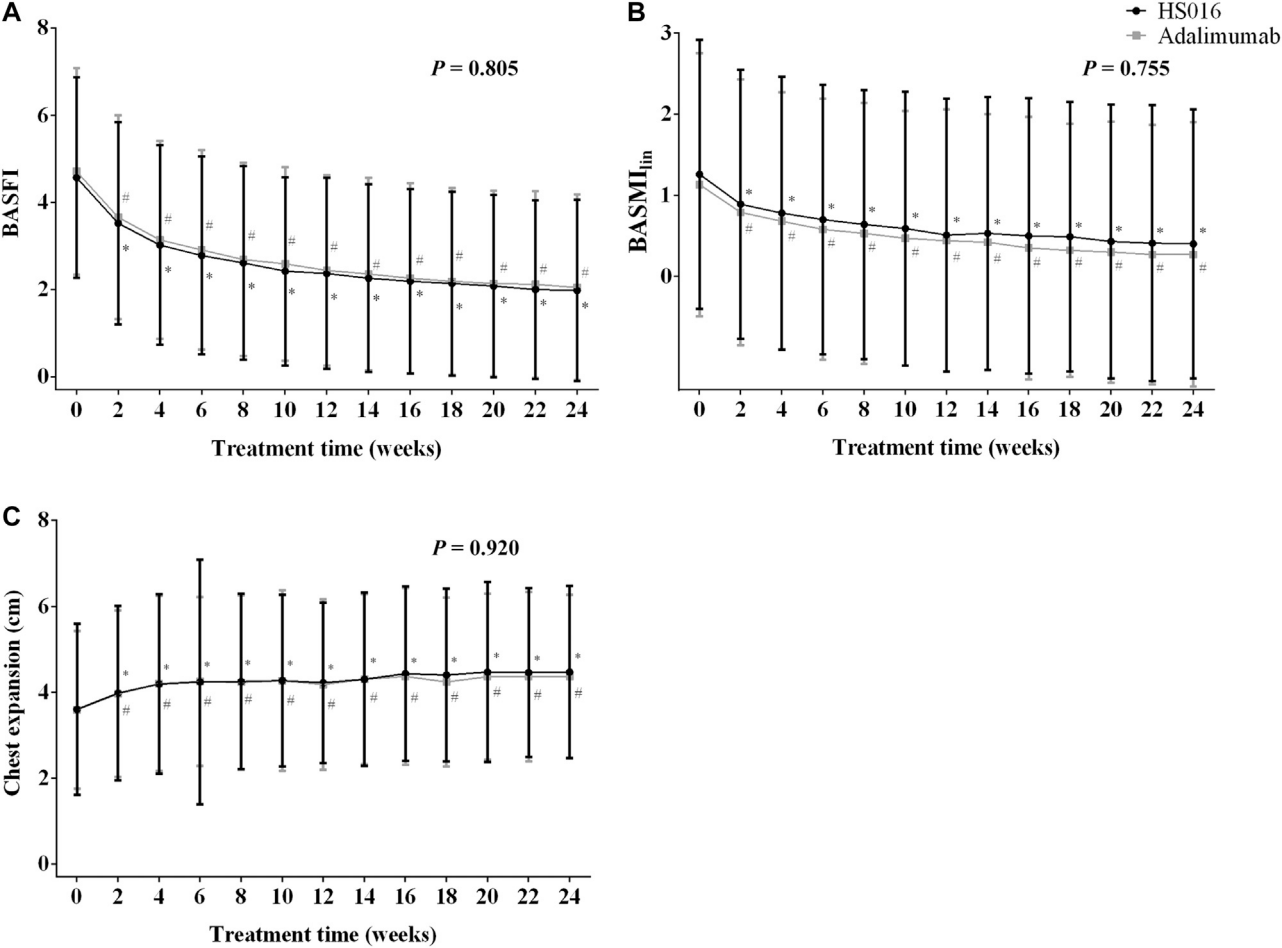
**(A)** BASFI, **(B)** BASMI_lin_, and **(C)** chest expansion at each time point during the 24-week treatment period in patients treated with HS016 or adalimumab. *p < 0.05, compared with baseline in the HS016 group; ^#^ *p* < 0.05, compared with baseline in the adalimumab group. BASFI, Bath ankylosing spondylitis functional index; BASMI_lin_, linear Bath ankylosing spondylitis metrology index.

The BASFI scores were significantly lower at all the time points analyzed compared to those at baseline in both treatment groups (all *p* < 0.05) (Figure 2A). The scores were apparently slightly higher in the adalimumab group at each time point, but statistical significance was not reached (*p* = 0.805) (Figure 2A). In fact, the changes from baseline were virtually identical in the two groups at week 2 (−1.05 ± 1.73 and −1.05 ± 1.56; −22.98 and −22.29%, in the HS016 and adalimumab groups, respectively), week 12 (−2.20 ± 2.08 and −2.27 ± 2.00; −48.14 and −48.20%) and week 24 (−2.59 ± 2.23 and −2.66 ± 2.11; −56.67 and 56.48%); but without statistical significance (all *p* > 0.05).

### Bath Ankylosing Spondylitis Metrology Index and Chest Expansion at Each Time Point

After 2 weeks of treatment, the BASMI_lin_ score had significantly decreased from 1.26 ± 1.66 to 0.89 ± 1.66 (*p* < 0.05) in the HS016 group (−0.37 ± 0.55; −29.37%) and from 1.13 ± 1.62 to 0.79 ± 1.64 (*p* < 0.05) in the adalimumab group (−0.33 ± 0.67; −29.20%) (Figure 2B; Table 2). The declining trend continued until week 12, at which point there were differences of −0.75 ± 0.79 (−59.52%) and −0.69 ± 0.82 (−61.06%) from baseline in the HS016 and adalimumab groups, respectively. The declining trend slowed from week 12 to week 24, at which point there were differences of −0.86 ± 0.91 (−68.25%) and −0.85 ± 0.91 (−75.22%) from baseline in the HS016 and adalimumab groups, respectively (Figure 2B). No significant differences were found with regard to the BASMI_lin_ scores at any time point between two groups *p* = 0.755).

Chest expansion increased from 3.60 ± 1.99 to 3.98 ± 2.03 cm in the HS016 group and from 3.59 ± 1.84 to 3.96 ± 1.94 cm in the adalimumab group at week 2 (10.83% and 10.31% compared with baseline, respectively) and continued to significantly increase until the end of the treatment period, at which point the difference from the baseline was 24.44 and 21.91%, respectively (all *p* < 0.05) (Figure 2C). The trends in chest expansion were approximately the same in the two groups during the initial 14 weeks of treatment, but the values in the HS016 group appeared to become higher than in the adalimumab group during the period from week 16 to week 24, but the results were not significantly different (*p* = 0.920).

## Discussion

Primary phase III randomized clinical trial data analysis has demonstrated that HS016 was comparable to adalimumab with regard to composite efficacy endpoints during a 24-week treatment period. A comparison of adverse events (AEs) produced by HS016 and adalimumab has been previously reported and revealed 1,573 treatment emergent AEs (TEAEs) among 352/416 (84.6%) patients in the HS016 group and 751 TEAEs in 200/232 (86.2%) patients in the adalimumab group. The majority of TEAEs were categorized as mild or moderate and the incidence of serious AEs (SAEs) was 4.3% for HS016 vs. 2.6% for adalimumab. TEAEs and SAEs rates between the two groups were not significantly different. Plasma concentrations, area under the plasma drug-concentration-time curve (AUC_τ_), steady-state maximal concentration (C_max,ss_) and other pharmacokinetic parameters were similar during the steady-state period for both drugs, regardless of the anti-drug antibody status (positive or negative). However, while a number of individual efficacy indicators were used to assess the above efficacy endpoints (Su et al., 2020), the changes in the individual indicator at various time points, which reflected the treatment effect in real time, remained unknown.

The results of the current subanalysis have revealed that the individual efficacy indicators, namely total and nocturnal back pain, SJC, MASES, PaGA, and PhGA of disease activity, BASDAI score, BASFI score, BASMI_lin_ score and chest expansion were virtually identical in the two treatment groups at each time point, indicating that HS016 has comparable efficacy to adalimumab. The large SDs of individual efficacy indicators in this study may be due to the uncertainty of essentially subjective evaluations in each patient and only major inequalities are detected. However, according to the guideline (Sieper et al., 2009), these assessments of efficacy indicators can better reflect the improvement degree of symptoms and signs in patients with AS, thus it also can be used to demonstrate the comparability between two groups.

In the present analysis, each of the individual efficacy indicators showed declining or increasing trends during treatment, and these improvements represented significant differences from the baseline values in both groups as early as week 2 after the start of treatment. This rapid onset of the effect of treatment with HS016 or adalimumab was also observed when the ASAS20 response rate (46.4% in HS016 and 47.4% in adalimumab) was evaluated in the original study (Su et al., 2020). These results are consistent with the previous adalimumab-placebo trial, in which the ASAS20/ASAS40 response rate, BASDAI score, nocturnal back pain and SJC were significantly improved after 2 weeks of treatment (Haibel et al., 2006; van der Heijde et al., 2006; Huang et al., 2014). The significant improvement in all indicators by week 12 revealed in the present analysis suggested that HS016 produced a clinically meaningful improvement in AS symptoms similar to that of adalimumab (van der Heijde et al., 2006; Huang et al., 2014) or other TNF-α inhibitors (Park et al., 2013). Furthermore, the slowed rates of decline/increase in these indicators from week 12 to week 24 suggested that the effects of HS016 occurred at an early stage during therapy and were then maintained during continued therapy. The results from a study of infliximab, a TNF-α inhibitor similar to adalimumab, suggested that these effects might persist for as long as 3 years (Braun et al., 2005).

The only differences between the two groups were found for changes in the total back pain score from baseline to weeks 10, 12, and 22 (all *p* < 0.05), when the change induced by adalimumab was significantly higher than that induced by HS106 indicating that adalimumab improved total back pain at a more rapid rate than HS016. We also found that in the adalimumab group, SJC at week 2 was not significantly lower than at baseline, which may be due to the larger standard deviation of the data (both at baseline and week 2).

The presented subanalysis had a number of limitations. Beside the short treatment and follow-up times, and the absence of MRI, peripheral arthritis, uveitis, enthesitis and other data, the primary phase III clinical trial was not predesigned to analyze these individual efficacy indicators as endpoints, which may have limited the power of the analysis; moreover, the number of multiple comparisons increased the chance of spurious significant associations. Therefore, we only compared the trends in these individual efficacy indicators at different time points that would provide the most meaningful guidance for clinical practice.

In conclusion, HS016 was similar to adalimumab in terms of total and nocturnal back pain, SJC, MASES, PaGA and PhGA of disease activity, BASDAI score, BASFI score, and the BASMI_lin_ score at each time point during the 24-week trial, which provided further insight into the efficacy of this treatment. Further long-term evaluation of HS016 treatments for AS patients are warranted.

## Data Availability Statement

The raw data supporting the conclusions of this article will be made available by the authors, without undue reservation.

## Ethical Statement

The studies involving human participants were reviewed and approved by the ethics committees of the participating centers. The patients/participants provided their written informed consent to participate in this study.

## Author Contributions

ML and XfZ contributed to the study conception and design. Material preparation, data collection and analysis were performed by all authors. The first draft of the manuscript was written by JS and LH; all authors commented on subsequent versions of the manuscript. All authors read and approved the final manuscript submitted to the journal.

## Funding

This work was supported by the Chinese National Key Technology R&D Program, Ministry of Science and Technology (No. 2017YFC0907601); Medicine and Health Technology Innovation Project, Chinese Academy of Medical Sciences (No. 2019-I2M-2-008) and ZHEJIANG HISUN PHARMACEUTICAL CO., LTD. The funder was not involved in the study design, collection, analysis, and interpretation of data, the writing of this article or the decision to submit it for publication.

## Conflict of Interest

The authors declare that this study received funding from ZHEJIANG HISUN PHARMACEUTICAL CO., LTD.
