# Supplementary figures and images for: Changes in Efficacy Indicators for Adalimumab Biosimilar Candidate (HS016) for the Treatment of Active Ankylosing Spondylitis at Various Time Points
Source: Front Pharmacol. 2020 Dec 7;11:606497. doi: 10.3389/fphar.2020.606497 (PMC7750525; doi:10.3389/fphar.2020.606497)

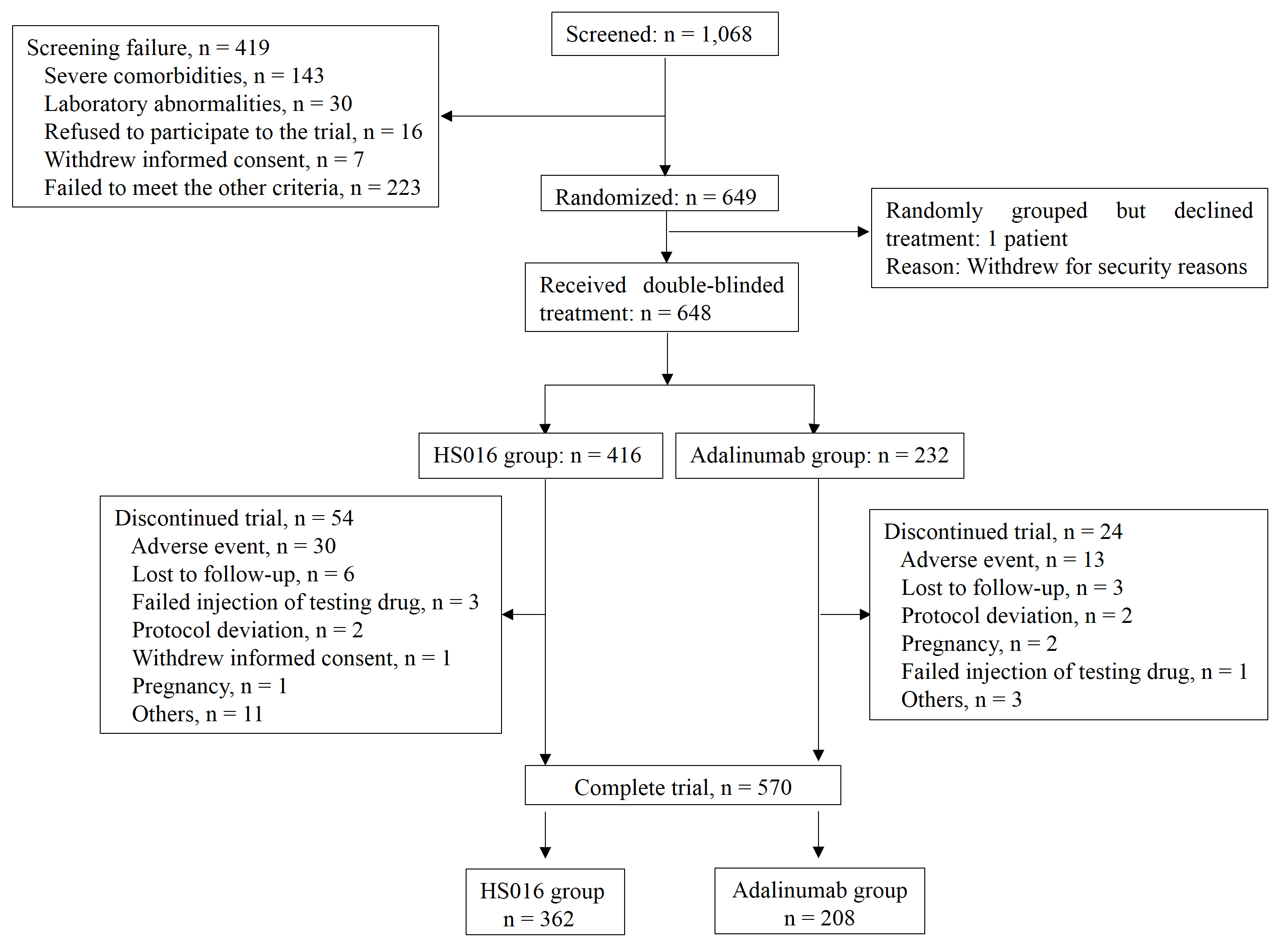

Supplement: Supplementary file 1 [file image1.tif]
